# An analysis of compassionate access programmes for novel oncology drugs

**DOI:** 10.1007/s11845-025-03930-7

**Published:** 2025-03-21

**Authors:** Sarah A. Kelly, Karine E. Ronan, Mohammed Zameer, Jennifer Brown, Grainne Johnston, Ruth Adams, Dearbhla Murphy, Deirdre Kelly, Waseem Darwish, John McCaffery, Geraldine O’Sullivan Coyne, Emily Harrold, Shahid Iqbal, Darren Cowzer, Austin G. Duffy

**Affiliations:** 1 School of Medicine, University College Dublin, Dublin, Ireland; 2 Department of Medical Oncology, Mater Misericordiae University Hospital, Dublin, Ireland; 3 University College Dublin Centre in Translational Oncology, Dublin, Ireland

**Keywords:** Approval, Cancer, Compassionate access, Drug, Immunotherapy, Ireland, Managed access, Novel, Oncology, Pharmacoeconomics, Reimbursement, Targeted therapy

## Abstract

**Background:**

Despite the rise in the number of approved novel oncology drugs, just over half of all new cancer medicines approved by the EMA between 2017 and 2021 were granted reimbursement in Ireland by the HSE. Compassionate access programmes (CAPs) are a means of providing managed access to drugs which are of proven benefit but have not yet received full approval for reimbursement by the state, or where the requested indication has not been yet been authorised/licensed.

**Methods:**

A retrospective review was performed of patients attending The Mater hospital for treatment of advanced malignancy who availed of a CAP between August 2012 and July 2022. Clinical data collected included disease type, treatment received, duration of treatment received, and best response to treatment. To categorize outcome “Clinical Benefit” was defined as a radiological complete, partial, or stable disease response to treatment.

**Results:**

One hundred and thirteen patients were included in the study. Ninety-three received at least one dose of CAP treatment. Treatment duration ranged from 0 to 112 months, with 12 patients on treatment for ≥ 2 years. *N* = 47 (42%) experienced a Clinical Benefit. Of these, *N* = 7 experienced a complete response [CR]. Thirty patients (27%) did not receive a planned treatment or died within 3 months of treatment.

**Conclusions:**

In this review of a decade of CAPs at our institution we observed that a significant proportion of patients derived a clinical benefit from CAP treatment. Unfortunately, however, a significant proportion of patients did not receive a planned treatment due to disease progression or died within 3 months of treatment suggesting availability came too late. While CAPs can provide meaningful benefit, they are not a substitute for timely approval of novel agents.

## Background

In the past decade, there has been a marked increase in the number of novel oncology drugs approved for use across Europe and the USA [1], reflecting great advances in the basic science underlying cancer and the drug development that emerges from this [2]. Providing equitable and timely patient access to these new therapies is not straightforward however, given the increasing costs of these medicines and the implications for wider society in terms of where public funding is best directed. From a total of over 300 anti-cancer drugs licensed by the European Medicines Agency (EMA) in the period from 2010 to 2019, approximately 100 of these drugs were approved for re-imbursement in Ireland [3]. Ireland is the second slowest country among the EU-15 to provide access to novel anti-cancer medicines with proven efficacy which have already been approved by the EMA. Irish patients wait on average twice the length of time to access these new medications than the EU-15 average [1].

Compassionate access programmes (CAPs) are a means by which clinicians can access effective and EMA-approved oncology drugs while awaiting a local/national decision on reimbursement in Ireland. These programmes arise when a pharmaceutical company make an approved medicine available to patients who fit the licensed indication in advance of re-imbursement being agreed. Also referred to as expanded or managed access programmes, oncologists can apply directly to pharmaceutical companies on behalf of their patients to access these treatments which are currently unavailable within the Irish healthcare system.

The aim of this study was to characterise the cohort of oncology patients in the Mater Misericordiae University Hospital (MMUH) who have availed of a drug via a CAP and to determine whether these patients experienced a clinical benefit from the treatment received, and assess the role and value of these programmes within the Irish health system. This is the first Irish data encompassing the individual clinical benefit of such programmes within Ireland.

## Methods

Patients attending MMUH for treatment of an advanced malignancy and who had enrolled in a CAP between August 2012 and July 2022 were identified for inclusion in our study, via a pharmacy-maintained database.

A retrospective review of paper and electronic medical records, including a comprehensive review of radiological and clinical data, was carried out for each patient using these variables. Data gathering was supported and verified using prescriptions within the BD Cato™ prescribing software to cross-check the treatments chosen for each patient and the durations of therapy received.

Radiology reports were reviewed to determine patient benefit or lack of benefit across time.

Due to the retrospective nature of the analysis, response evaluation criteria in solid tumours (RECIST) could not be applied. Instead, the contemporaneous radiology report was used to determine whether a patient had experienced a clinical benefit from EAP treatment. A clinical benefit was defined as a patient’s target lesions on imaging receding completely (complete response), reducing in size (partial response) or remaining stable in size (stable disease) while receiving treatment. Conversely, a lack of clinical benefit was observed when a patient’s target lesions increased in size, or when a patient was unable to receive a planned CAP treatment due to disease progression or death prior to the treatment start date.

Descriptive statistics and quantitative crosstabulations were generated on variables gathered using the Statistical Package for the Social Sciences (SPSS) software. A waterfall plot and Kaplan–Meier curve were generated using the Statistical Analysis System (SAS) software. A swimmer plot was generated utilising Microsoft Excel.

This study was granted approval by Mater Misericordiae University Hospital Clinical Audit and Effectiveness Committee in February 2022.

## Results

A total of 113 patients received approval for a CAP treatment in MMUH from August 2012 until July 2022 and were identified for inclusion in this study. Ninety-three of these patients proceeded to receive CAP treatment. The median age was 65 years (range 29–94). All patients who received a CAP treatment had a diagnosis of stage IV malignancy. Patient characteristics are summarised in Table 1.

**Table 1:** Baseline characteristics of patients who received approval for CAP treatment in MMUH

Patient characteristics	*n* = 113
Female gender, no. (%)	51 (45.1)
Age in years, median (range)	65 (29–94)
Patients who received treatment with alternative treatment lines prior to compassionate access treatment	95
Patients who received ≥ 2 previous treatment lines	65
Cancer diagnosis – no. (%)	
Prostate	19 (16.8)
Breast	14 (12.4)
Non-small cell lung	14 (12.4)
HCC	9 (8.0)
Endometrium	9 (8.0)
Colon	9 (8.0)
Gastric	7 (6.2)
Clear cell renal	7 (6.2)
Papillary renal	6 (5.3)
Non-uveal melanoma	5 (4.4)
Small cell lung	2 (1.8)
Follicular dendritic cell	2 (1.8)
Ovary	2 (1.8)
Penis	2 (1.8)
Thymoma	1 (0.9)
Basal cell	1 (0.9)
Gallbladder	1 (0.9)
Mesothelioma	1 (0.9)
Oesophageal	1 (0.9)
Angiosarcoma	1 (0.9)
CAP treatment approved – no. (%)	
Nivolumab	42 (37.2)
Atezolizumab	19 (16.8)
Enzalutamide	11 (9.7)
Alpelisib	8 (7.1)
Apalutamide	6 (5.3)
Cabazantinib	5 (4.4)
Dabrafenib + Trametinib	5 (4.4)
Pembrolizumab	4 (3.5)
Neratinib	3 (2.7)
Ceritinib	2 (1.8)
Ipilimumab	2 (1.8)
Talazobarib	2 (1.8)
Alectinib	1 (0.9)
Avelumab	1 (0.9)
Lorlatinib	1 (0.9)
Vemurafenib	1 (0.9)

**Fig. 1:**
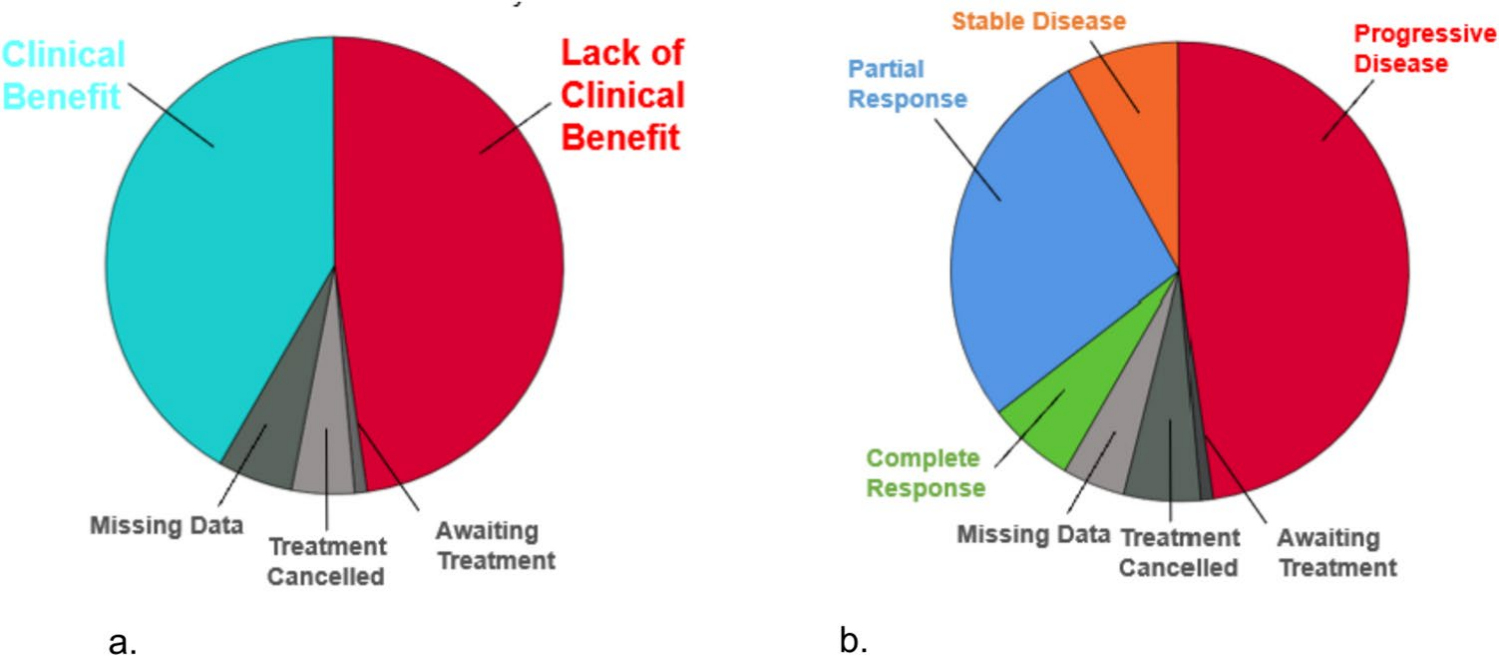
Pie charts summarising patient outcomes on CAP treatment. **A** Forty-two percent of patients experienced a clinical benefit from CAP treatment. *Treatment cancelled* represents those patients whose treatment was cancelled due to pre-existing contraindications to therapy that came to light prior to initiation (*N* = 6, 5%). One patient was awaiting to begin a planned treatment at the time of data collection. Data was missing in 5 cases (4%). Reasons for missing data included lack of documentation of treatment choices, durations of treatment, and patient outcomes consistently across time. **B** A further breakdown of clinical benefit by response to treatment

Forty-seven patients (42%) in our study experienced a clinical benefit from a CAP treatment. Seven patients experienced a complete response [CR] with 6 of these patients exhibiting a sustained CR at the cut-off of data collection. Thirty-one patients experienced a partial response [PR] to a CAP treatment and 9 patients experienced stable disease [SD]. Fifty-four patients experienced a lack of clinical benefit, or progressive disease [PD]. Of note, this parameter includes those patients who were ultimately unable to receive a planned CAP treatment due to clinical progression or death prior to commencing treatment. It was deemed beneficial to include these patients in our analysis, as patients who had CAP treatment planned but did not ultimately receive it due to the progression of time, also failed to see a benefit from the CAP, despite enrolment. Five patients had data missing in regards to their best response to CAP treatment, while 7 patients did not receive CAP treatment for reasons other than clinical progression or death. Patient outcomes on CAP treatment are summarised in Fig. 1.

**Fig. 2:**
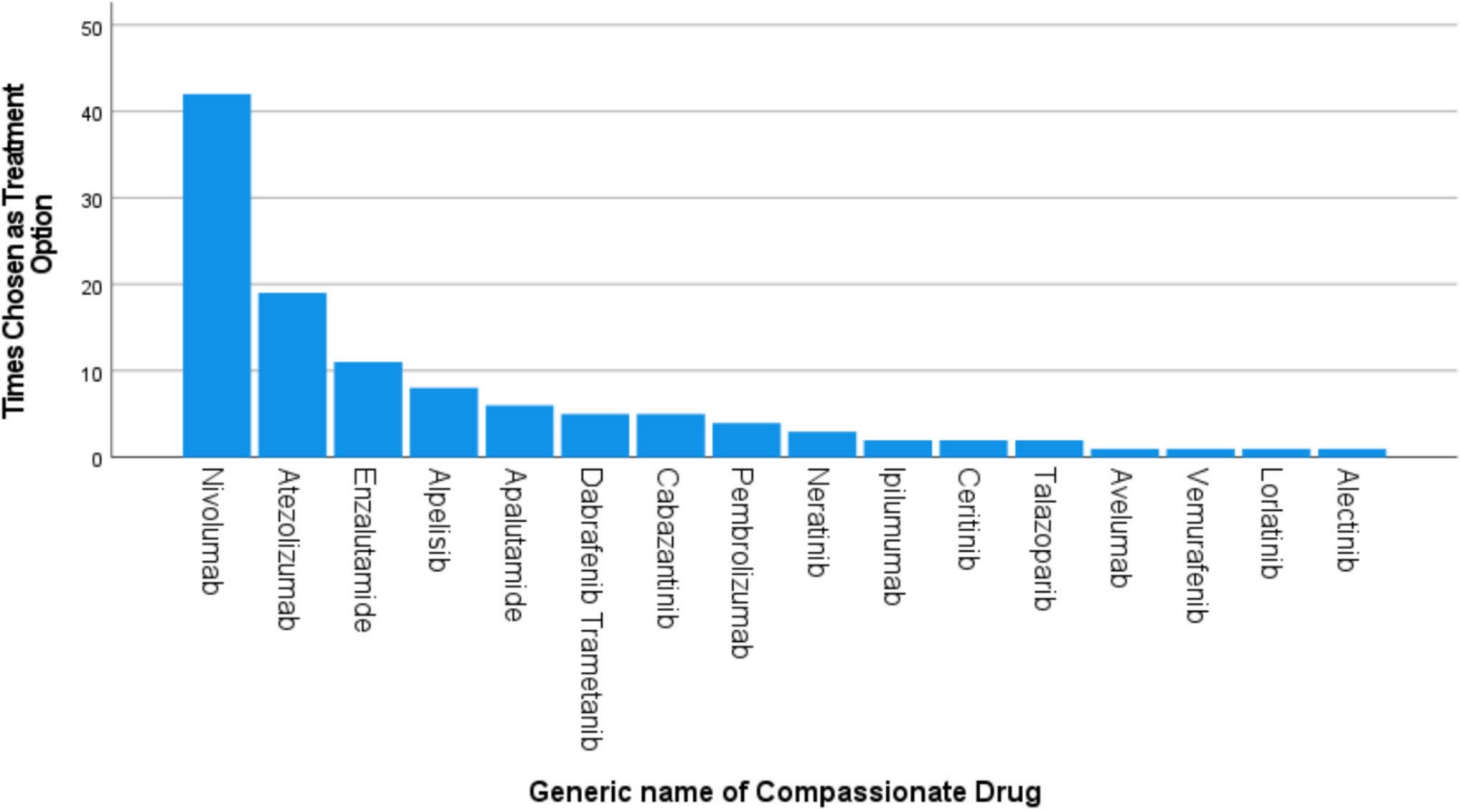
Summary of CAP treatments received. Nivolumab was the most frequently accessed drug via CAP, followed by Atezolizumab and Enzalutamide

**Fig. 3:**
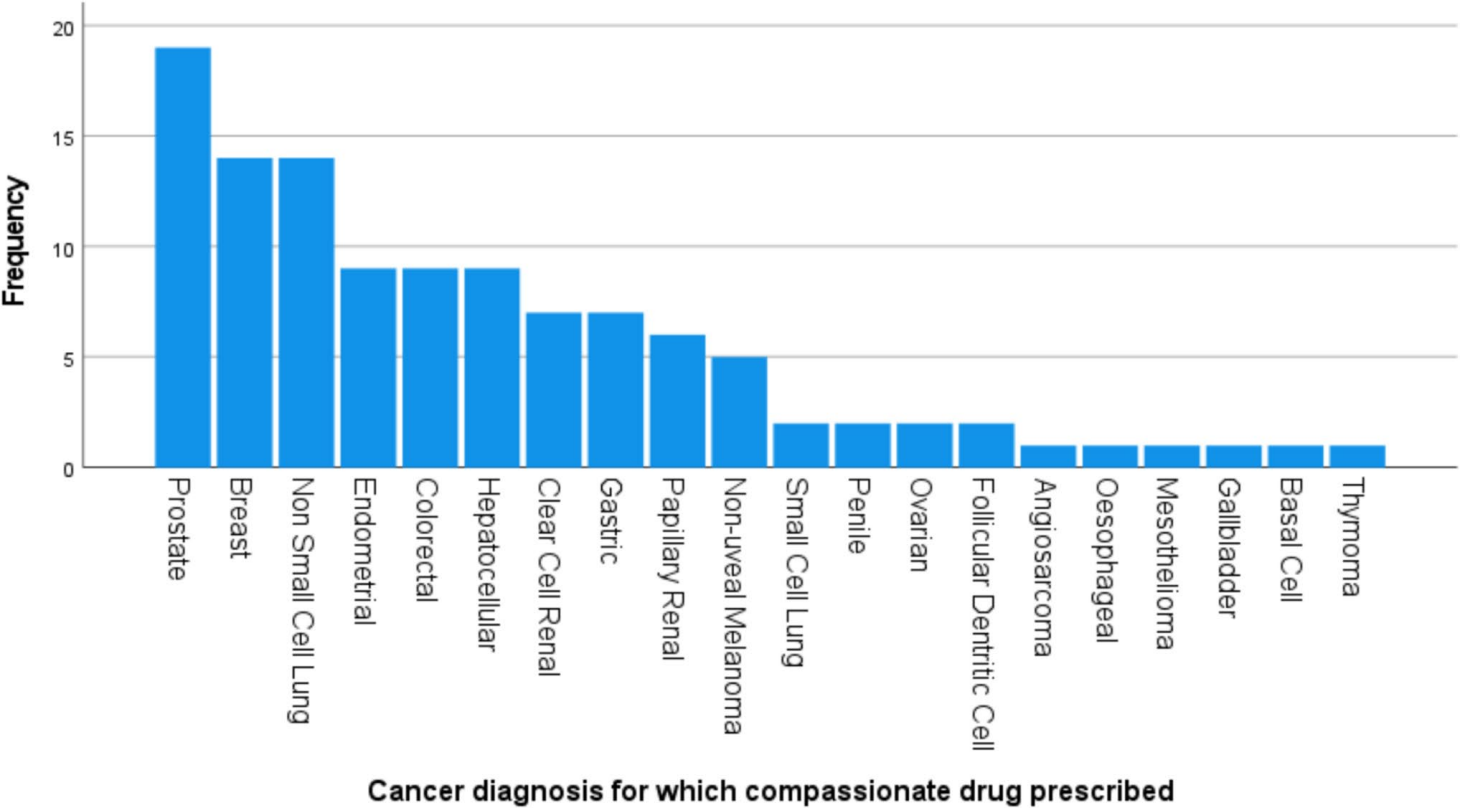
Graph outlining the number of patients based on cancer diagnosis in the study population. Prostate cancer and breast cancer were the most frequent cancer subtypes to avail of CAP treatments in our study, followed by non-small cell lung cancer and endometrial cancer

Ninety-three patients received at least one cycle of CAP treatment, with a total of 16 different treatments received, summarised in Fig. 2. Most CAP treatments received were immunotherapy-based treatment (60%), as well as anti-androgen (15%) and small molecule targeted therapies (25%)*.* A breakdown of patients by cancer diagnosis is outlined in Fig. 3.

**Fig. 4:**
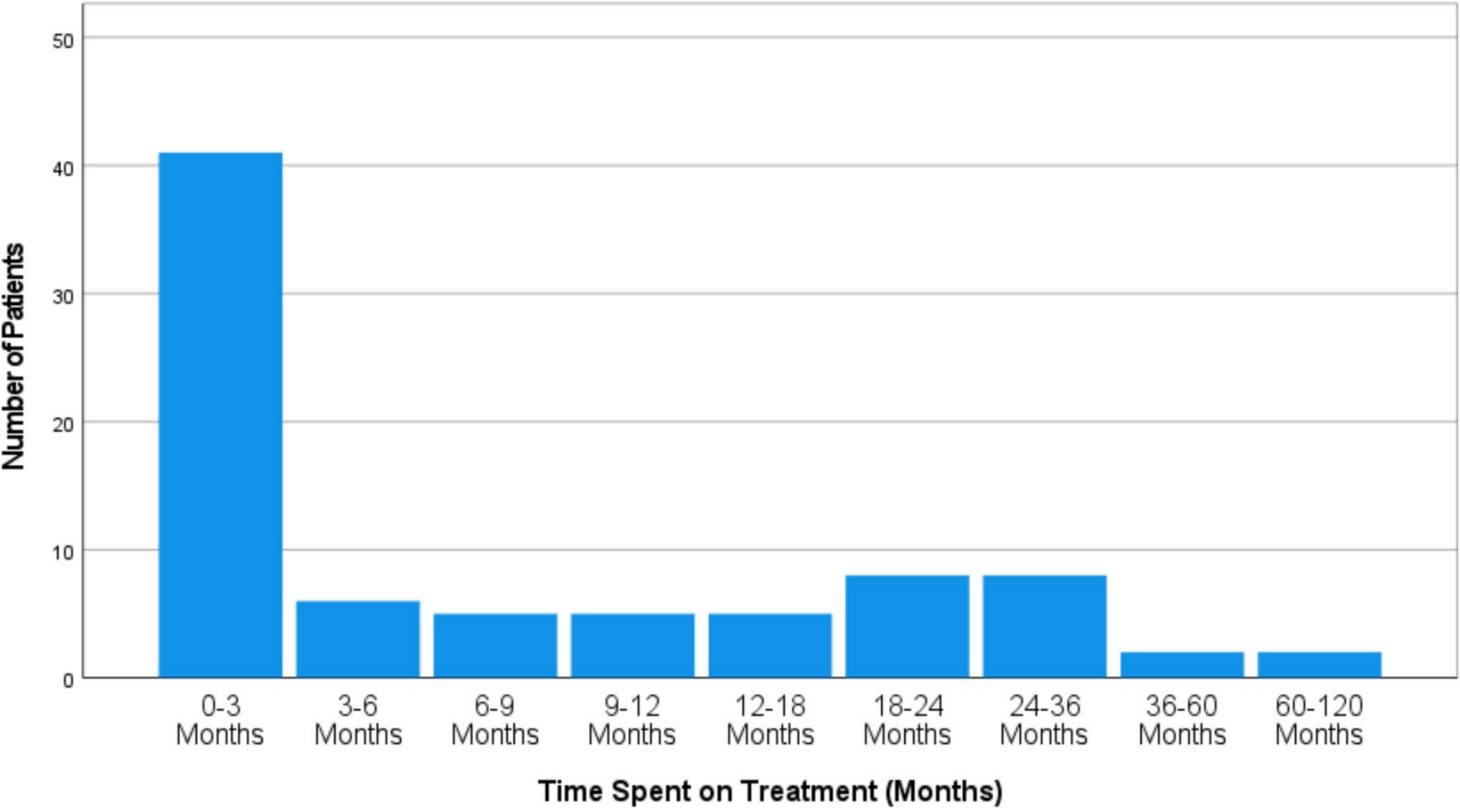
This graph outlines the duration of CAP treatment. Most patients received treatment for ≤ 3 months. A small proportion of treatment had received treatment for 120 months at time of data collection

Treatment durations ranged from 0 to 112 months, with 12 patients receiving treatment for more than two years. There was considerable variability in treatment duration, as shown in Fig. 4.

**Fig. 5:**
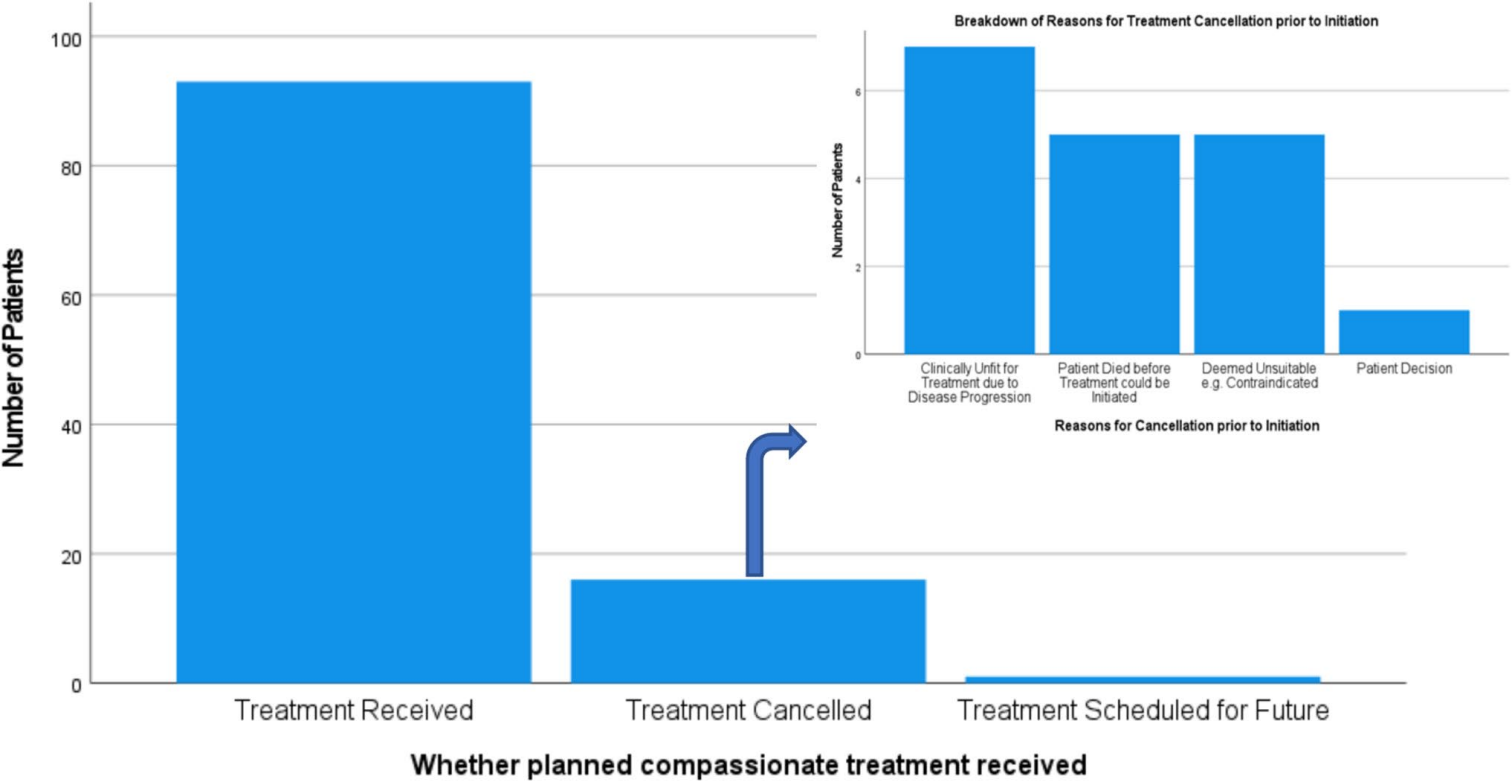
Graph outlining the reasons for treatment cancellation prior to initiation. The majority of patients did not receive a planned CAP treatment due to disease progression

Twelve patients (10.6%) for whom a CAP treatment was approved, did not receive the planned treatment due to clinical progression or death. As stated, these patients are included in the lack of clinical benefit figure. Reasons for treatment cancellation are outlined in Fig. 5.

**Fig. 6:**
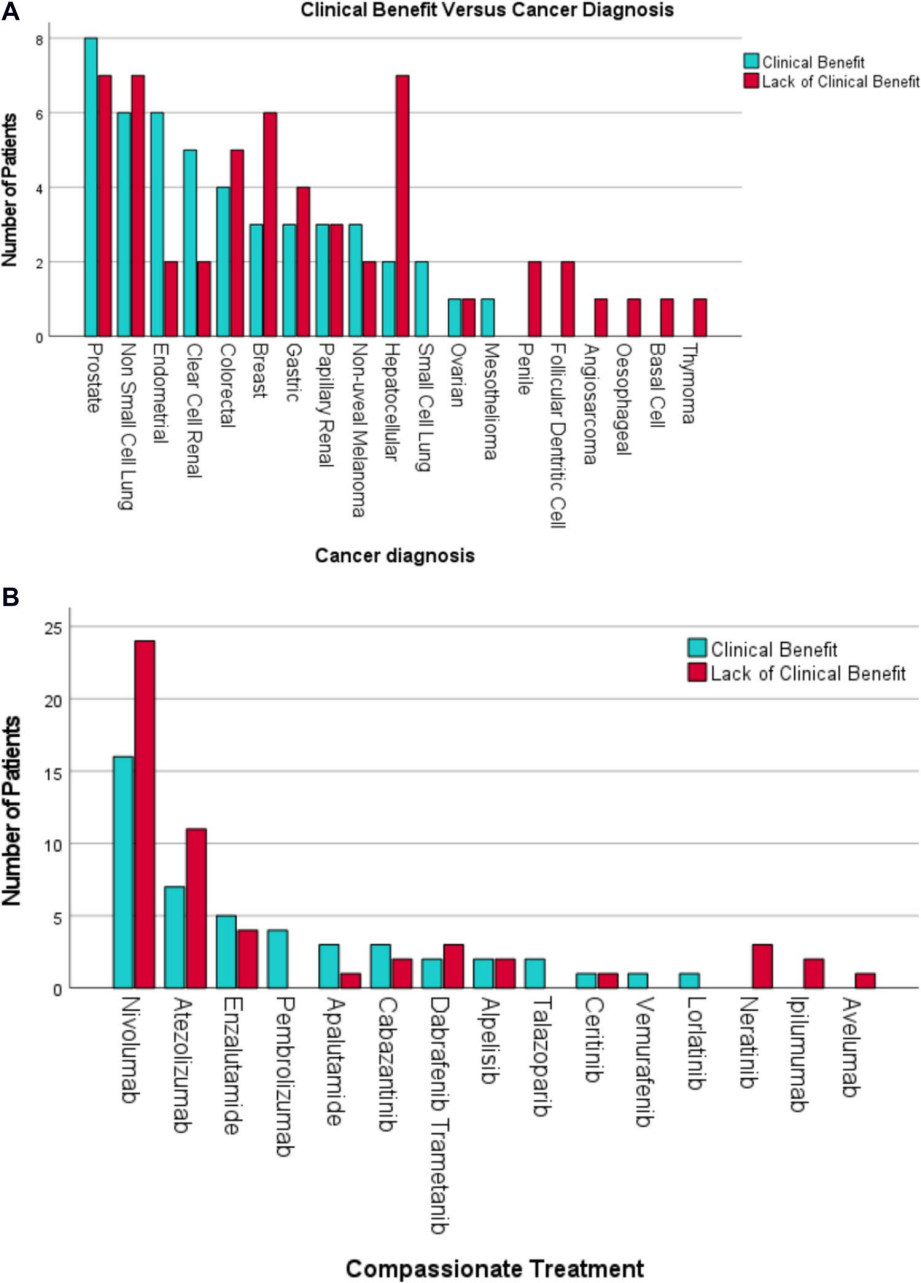
**A** Graph outlining patient outcomes according to cancer diagnosis. **B** Graph outlining patient outcomes based on CAP treatment received. Note again that lack of clinical benefit figure includes those patients that were unable to receive a planned CAP treatment due to clinical progression, or death

For the patients that did receive CAP treatment (*N* = 93), the most frequent reason for treatment discontinuation was disease progression (*n* = 49), followed by toxicity (*n* = 14) and end of prespecified treatment availability as per CAP (*n* = 6). A substantial number of patients with prostate cancer, endometrial cancer, and renal cell carcinoma experienced a clinical benefit from CAP treatment. Figure 6 outlines patient outcomes based on CAP treatment received and by cancer diagnosis*.*

**Fig. 7:**
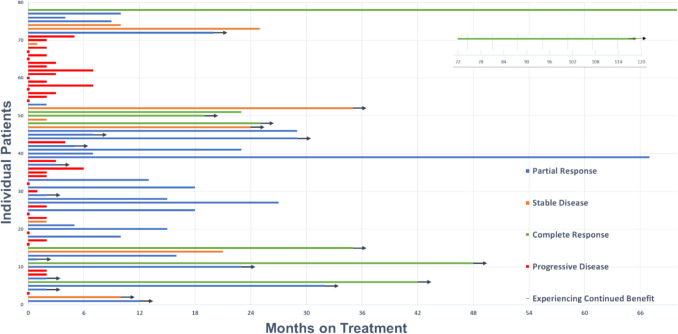
Swimmer plot representing the duration of treatment for each patient as well as best response to CAP treatment. Arrowheads were utilised to represent those patients that were continuing to show this response at the time of data collection, with 6 patients continuing to show a sustained complete response

A swimmer plot, Fig. 7, represents the duration of CAP treatment for each patient as well as best response treatment. Fourteen patients who received treatment are not represented in the swimmer plot due to incomplete documentation of number of treatments received.

**Fig. 8:**
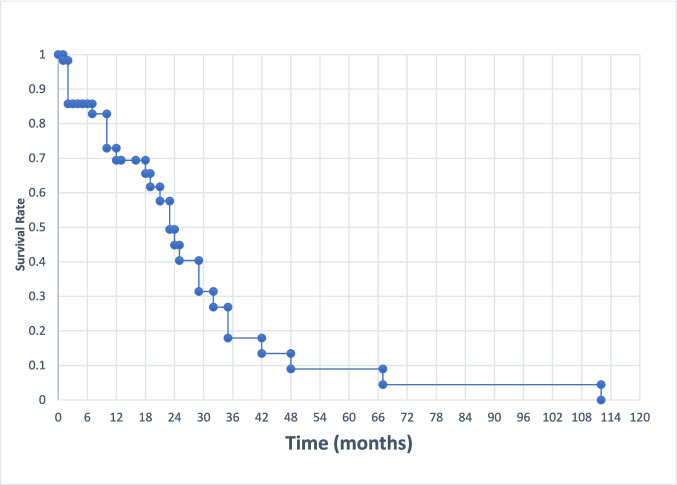
Kaplan–Meier curve representing survival for patients included in our study and for whom complete survival data was available, *n* = 70

The survival rate at 12 months in our study population was 70%, as outlined in Fig. 8. Median survival in the study population (*n* = 70) was 24 months. Patients who had no clear death date documented, who were lost to follow up (*N* = 9), or who had unclear treatment durations documented (*N* = 14) were excluded in this analysis.

## Discussion

Compassionate access programmes (CAPs) are a means of providing access to drugs which have already been approved by regulatory authorities, but which have not yet been approved for state reimbursement. These programmes, similar to the clinical trials which preceded them and led to their approval, reflect the evolution in drug development and offer the latest and frequently best therapeutic option for a patient. As the pace of both scientific advances and drug prices increase, it has become a genuine challenge for national authorities to provide timely access to novel and effective drugs within the context of multiple competing demands and unmet needs.

This study focused on oncology drugs and reviewed the use of CAPs at our institution over a 10-year period, among patients with advanced cancer. Over a hundred patients were prescribed an EMA-approved anticancer drug in this manner with 42% deriving a clinical benefit as defined radiologically. This is a substantial benefit considering the advanced nature of their cancer and multiple previous lines of treatment received by the majority of the patients in our study. This benefit is reflected in our survival analysis, which found a survival rate of 70% at 12 months, and a median survival of 24 months among our study population. Seven patients in our study were reported to experience a complete response to a CAP drug, with six of these patients continuing to experience a sustained complete response following completion of the CAP treatment regimen.

While it is beyond the scope of this study to determine drug efficacy, all the drugs accessed via CAPs were granted EMA approval based on data from randomized clinical trials and the decision to treat the patient was conditional upon their matching the labelled indication.

Providing timely access to new medicines is a complex issue for all societies and each country and health system faces the constraints of their own particular set of circumstances. Unfortunately in Ireland the situation is particularly grave for patients, as outlined by Hofmarcher et al., who reported that just over half of all new cancer medicines approved by the EMA between 2017 and 2021 were reimbursed by the HSE at the beginning of 2022 [1]. Ireland places behind almost all other EU-15 countries in terms of time to new drug availability. When compared to countries with the quickest drug approval processes such as Germany, with an average time to oncology drug availability of 100 days, Ireland lags very far behind at 661 days [1, 4]. This report details cancer survival rates in Ireland, particularly for breast and ovarian cancer patients, which also lags far behind other EU-15 countries [5, 6]. It is well documented in this publication that there is no specific data available on spending on cancer care in Ireland, making assessment of cost versus benefit for cancer care challenging [1]. Data from the Waiting to Access Innovative Therapies (W.A.I.T.) indicator survey 2022 found that Ireland falls well below the EU average both in terms of the rate of anticancer drug availability, and time to availability of anticancer drugs [4]. In real terms, this translates to Irish cancer patients waiting almost 2 years to access licensed and effective drugs.

One such example of a drug that encountered a significant lag time prior to becoming available for Irish patients is Osimertinib. This targeted therapy, effective in the treatment of EGFR mutant non-small cell lung cancer, significantly improved overall survival in a clinical trial by over 8 months for patients with advanced disease when compared with older generation tyrosine kinase inhibitors [7]. Osimertinib received full FDA approval in March 2017; however, it only became available to Irish patients in July 2020 [8].

While almost half of the patients in our study did not experience a clinical benefit from CAP treatment, the reasons for this were multifactorial, including lack of efficacy. But the finding that 10.6% of patients did not actually receive the planned CAP treatment due to clinical progression or death prior to treatment start date, and that 16% of patients died within 3 months of receiving the CAP treatment suggests that the CAP treatment was initiated too late in the disease course.

There are several limitations to our study which generally reflect the retrospective nature of the analysis with dependence on the contemporaneous medical record and radiology reports. Prospective assessment via the RECIST criteria could not be employed. In addition, given that the treatment took place outside of a clinical trial drug toxicity and adverse events were not systematically recorded. We are therefore unable to comment on the real-world application as it pertains to tolerability of the patients experiencing a clinical benefit nor assess the impact of treatment upon quality of life nor assess toxicity.

The issue of timely access to drugs is complicated, particularly for centralized publicly funded health systems, this is acknowledged by the *National Cancer Strategy 2017–2026* [9]. Directing funding to one area is a decision that inevitably disadvantages another. The incorporation of Early Access as a Key Performance Indicator within the HSE indices of cancer care should be prioritised. Lengthy reimbursement discussions and pricing negotiations need to be refined and redesigned for subgroups of patients with a high medical need. Separation of this process from treatment for chronic conditions is one potential suggestion and is something which is already being carried out in both the United Kingdom and France [10]. Novel reimbursement models such as ‘payment upon benefit’ or ‘coverage with evidence development’, whereby a new medicine is reimbursed for a limited period of time during which real-world data on its effectiveness are collected in clinical practice [11, 12], should be considered. Ireland is among a minority of EU-15 countries whereby there is limited to no data currently available on direct cost of cancer care and total health expenditure on cancer care and therapeutics [13]. Above all though there should be increased transparency throughout the approval process with published timelines and negotiation or pricing decisions and wider involvement of key stakeholders such as patients and doctors.

Similar recommendations are echoed in the report on the 2020 Mazars Review regarding the HSE reimbursement and pricing decision-making process, both of which were published in 2023, following completion of this study [14, 15]. Most notably enhanced transparency and improved patient participation in the process were emphasised throughout the review. This study further works to build on the recommendations offered and highlights how implementation of the same are essential to pave the way towards a more streamlined reimbursement process in our country.

Overall, while we observed that CAPs can provide a meaningful benefit for the patients that can avail of them, they are ultimately not a substitute for the timely approval of effective, clinical trial-validated therapeutics. While novel treatments come at a significant cost, so too does a lengthy reimbursement process.

## Data Availability

Data utilised in this manuscript is stored on a secure database within the MMUH network to protect patient privacy.
